# Soluble αKlotho downregulates Orai1-mediated store-operated Ca^2+^ entry via PI3K-dependent signaling

**DOI:** 10.1007/s00424-020-02510-1

**Published:** 2021-01-02

**Authors:** Ji-Hee Kim, Eun Young Park, Kyu-Hee Hwang, Kyu-Sang Park, Seong Jin Choi, Seung-Kuy Cha

**Affiliations:** 1 Department of Physiology, Yonsei University Wonju College of Medicine, 20 Ilsan-ro, Wonju, Gangwondo 26426 Republic of Korea; 2 Department of Global Medical Science, Yonsei University Wonju College of Medicine, Wonju, Republic of Korea; 3 Mitohormesis Research Center, Yonsei University Wonju College of Medicine, Wonju, Republic of Korea; 4 Institute of Mitochondrial Medicine, Yonsei University Wonju College of Medicine, Wonju, Republic of Korea; 5 Department of Obstetrics and Gynecology, Yonsei University Wonju College of Medicine, 20 Ilsan-ro, Wonju, Gangwondo 26426 Republic of Korea

**Keywords:** SOCE, STIM1, FGF23, CRAC channel

## Abstract

αKlotho is a type 1 transmembrane anti-aging protein. αKlotho-deficient mice have premature aging phenotypes and an imbalance of ion homeostasis including Ca^2+^ and phosphate. Soluble αKlotho is known to regulate multiple ion channels and growth factor-mediated phosphoinositide-3-kinase (PI3K) signaling. Store-operated Ca^2+^ entry (SOCE) mediated by pore-forming subunit Orai1 and ER Ca^2+^ sensor STIM1 is a ubiquitous Ca^2+^ influx mechanism and has been implicated in multiple diseases. However, it is currently unknown whether soluble αKlotho regulates Orai1-mediated SOCE via PI3K-dependent signaling. Among the Klotho family, αKlotho downregulates SOCE while βKlotho or γKlotho does not affect SOCE. Soluble αKlotho suppresses serum-stimulated SOCE and Ca^2+^ release-activated Ca^2+^ (CRAC) channel currents. Serum increases the cell-surface abundance of Orai1 via stimulating vesicular exocytosis of the channel. The serum-stimulated SOCE and cell-surface abundance of Orai1 are inhibited by the preincubation of αKlotho protein or PI3K inhibitors. Moreover, the inhibition of SOCE and cell-surface abundance of Orai1 by pretreatment of brefeldin A or tetanus toxin or PI3K inhibitors prevents further inhibition by αKlotho. Functionally, we further show that soluble αKlotho ameliorates serum-stimulated SOCE and cell migration in breast and lung cancer cells. These results demonstrate that soluble αKlotho downregulates SOCE by inhibiting PI3K-driven vesicular exocytosis of the Orai1 channel and contributes to the suppression of SOCE-mediated tumor cell migration.

## Introduction

*Klotho* is an aging-suppressor gene that encodes type 1 transmembrane glycoprotein called αKlotho [22, 23]. *Klotho*-deficient (*kl/kl*) mice show accelerated aging phenotypes with a severe imbalance of ion homeostasis including Ca^2+^ and phosphate (P_*i*_) [22, 24, 36]. The Klotho family comprises three members: αKlotho (encoded by the α*Klotho* gene; also known as *KL*), βKlotho (encoded by the *βKlotho* gene; also known as *KLB*), and γKlotho (encoded by *Lctl* gene; also known as *KLG*) [19, 20]. αKlotho has at least two functional modes including full-length membrane-bound form and soluble form. The membranous form of αKlotho binds to multiple fibroblast growth factor (FGF) receptors that function as an obligatory coreceptor for FGF23 to regulate P_*i*_ and Ca^2+^ homeostasis [7, 24, 36]. The extracellular domain of αKlotho is cleaved off and released into blood, urine, and cerebrospinal fluid to function as paracrine and/or endocrine hormone [19, 23]. This soluble form of αKlotho exerts aging suppression and organ protection with pleiotropic action including regulation of ion channels and growth factor signaling [19–21].

Soluble αKlotho can positively or negatively regulate transient receptor potential (TRP) superfamily of cation channels. αKlotho upregulates multiple TRPV channels including TRPV2, 5, and 6 [6, 26, 27], whereas several TRPC channels such as TRPC1, 3, and 6 are downregulated by αKlotho [9, 16, 25, 40, 42, 43]. Additionally, αKlotho positively regulates multiple K^+^ channels such as ROMK, Kv1.3, KCNQ1/KCNE1, and hERG channels [1, 2, 4, 29]. Soluble αKlotho increases the cell-surface abundance of TRPV and K^+^ channels by modifying their *N*-glycan through sialidase or β-glucuronidase activity of αKlotho [1, 2, 4, 6, 26, 27, 29]. This *N*-glycan modification by αKlotho increases the resident time of these channels at the plasma membrane by delaying their endocytosis [4, 27]. Conversely, αKlotho downregulates TRPC channels with a distinct mechanism. Soluble αKlotho inhibits TRPC1-mediated Ca^2+^ influx via binding directly to vascular endothelial growth factor receptor-2 (VEGFR2)/TRPC1 complex to promote their co-internalization [25]. αKlotho decreases the cell-surface abundance of TRPC6 and TRPC3 via inhibiting PI3K-dependent exocytosis of these channels [16, 42]. Recently, it is reported that soluble αKlotho targeting α2-3-sialyllactose binds to monosialogangliosides in lipid rafts to regulate TRPC6 [9, 41]. Overall, these studies provide compelling evidence suggesting that soluble αKlotho can regulate multiple ion channels via distinct mechanisms.

The ubiquitous second messenger Ca^2+^ regulates various cellular behaviors. Store-operated Ca^2+^ entry (SOCE) is vital for the maintenance of endoplasmic reticulum (ER) Ca^2+^ stores at precise levels for signaling in both non-excitable and excitable tissues to regulate a variety of cellular functions [31, 32]. The molecular components of SOCE are Orai1 and STIM1 (stromal interaction molecule 1), a pore-forming subunit, and an ER Ca^2+^ sensor, respectively. STIM1 is oligomerized and translocated to the plasma membrane during ER Ca^2+^ depletion that thereby triggers Ca^2+^ entry via Orai1, a Ca^2+^-selective channel at the plasma membrane [31, 32]. SOCE is a downstream effector of growth factor signaling. The explicit mechanism of Orai1 activation by PI3K-driven growth factor signaling in physiological conditions remains elusive. Moreover, soluble αKlotho suppresses aging and protects multiple disease progression by regulating growth factor signaling [19, 23]. The mechanism linking αKlotho and SOCE by growth factor signaling has not yet been identified. Here, we examined the mechanism by which soluble αKlotho regulates Orai1-mediated SOCE by growth factor stimulation and its functional implications.

## Materials and methods

### Materials and DNA constructs

2-(4-morpholinyl)-8-phenylchromone (LY294002) (cat no. 19-142) was purchased from Calbiochem (San Diego, CA, USA) and wortmannin (WMN) (cat no. W1628), brefeldin A (BFA) (cat no. B7651), and tetanus toxin A (TeNT) (cat no. T3194) were purchased from Sigma-Aldrich (St Louis, MO, USA). Recombinant αKlotho (human) protein was provided from R&D Systems (cat no. 5334-KL-025, Minneapolis, MN, USA). Non-targeting control oligonucleotides (cat. n. SN-1003) and small interfering RNA (siRNA) against human Orai1 (cat. n. M-014998-01-0005) were obtained from Bioneer (Daejeon, Korea) and Horizon Discovery Ltd. (Cambridge, UK), respectively.

Expression vectors for the transmembrane full-length mouse αKlotho (KLFL), an extracellular domain of mouse αKlotho (KL△TM), βKlotho, and γKlotho was a kind gift from Prof. Makoto Kuro-o (Jichi Medical University, Japan) [11, 24, 30]. Orai1 (mCherry-3xFlag-Orai1) and STIM1 (YFP-STIM1) plasmids were kindly provided from Drs. Joseph Yuan (University of North Texas, USA).

### Cell culture and transfection

A HEK293 cell line with an inducible mCherry-STIM1-T2A-Orai1-eGFP (provided from Dr. Chan Young Park (UNIST, Korea)) [34] and HEK293FT cells were cultured under high glucose DMEM medium (cat no. SH30243, Hyclone, Logan, UT, USA) supplemented with 10% fetal bovine serum (FBS) and 1% penicillin. The human breast cancer cell line MDA-MB231 and the human lung cancer cell line H1693 cells were cultured under RPMI1640 (cat no. SH30027, Hyclone, Logan, UT, USA) supplemented with 10% fetal bovine serum (FBS) and 1% penicillin.

All DNA plasmids were transfected by using X-tremeGENE HP DNA transfection reagent^Ⓡ^ (Roche, Mannheim, Germany) following the manufacturer’s instructions. Experiments were conducted 48 h after transfection. For knockdown by siRNA, oligonucleotides were transfected into MDA-MB231 and H1693 cells with DharmaFect (cat no. T-2001-03, Horizon Discovery Ltd., Cambridge, UK) following the manufacturer’s instructions. Cells were trypsinized, and re-seeded on the poly-lysine coated coverglasses after 48 h for live-cell Ca^2+^ imaging or on the 6-well plate for in vitro wound-healing assay. Experiments were conducted after 24 h re-seeding the cells.

### Real-time quantitative PCR analysis

Purified total RNA was extracted from the trypsinized pellets of HEK293FT cells through Hybrid-RTM total RNA purification kit (cat. n. 305-101, GeneAll, Seoul, South Korea) according to the manufacturer’s instructions. Complementary DNA (cDNA) was synthesized from 1 μg of total RNA by using a ReverTraAce® qPCR RT Master Mix with gDNA Remover (cat. n. FSQ-301, Toyobo, Osaka, Japan). The mRNA abundance was analyzed by real-time quantitative PCR with SYBR Green (cat. n. 204143, Qiagen, Germantown, MD, USA) using the following sequence-specific human primers: *ORAI1*, forward (F) 5′-TTGAGCCGCGCCAAGCTTAAA-3′, reverse (R) 5′-CATTGCCACCATGGCGAAGC-3′; *ORAI2*, F-5′-AAGTGCTTGGATGCGGTGCTG-3′, R-5′-GGAGCCAGGCAGGTCATTTATACG-3′; *ORAI3*, F-5′-TCAGCCGGGCCAAGCTCAAA-3′, R-5′-CATGGCCACCATGGCGAAGC-3′; *STIM1*, F-5′-GTACACGCCCCAACCCTGCT-3′, R-5′-AGGCTAGGGGACTGCATGGACA-3′; *STIM2,* F-5′-TGGACCTCTAACACGCCCACCT-3′, R-5′-CTGCGTATAAGCAAACCAGCAGCC-3′. For the analysis of each gene expression, the experiments were performed in triplicate in a real-time PCR system (7900HT, Thermo Fisher Scientific). Data were analyzed following the 2^-ΔΔCt^ method with 18S as the reference gene.

### Intracellular Ca^2+^ ([Ca^2+^]_*i*_) measurement

Intracellular Ca^2+^ concentration ([Ca^2+^]_*i*_) measurement was previously described [5]. A normal physiological salt solution was used for bath solution that contained (in mM) 135 NaCl, 5 KCl, 1 MgCl_2_, 2 CaCl_2_, 10 HEPES, and 10 glucose (pH 7.4). Fura-2 signals were obtained by alternating excitation at 340 or 380 nm, and detecting emission at 510 nm. Data acquisition and analysis were performed using the MetaFluor (Sutter Instruments, Novato, CA, USA) software. All [Ca^2+^]_*i*_ measurements were performed at ∼ 37 °C.

### Electrophysiological recordings

For recording Ca^2+^release-activated Ca^2+^ (CRAC) currents, HEK293FT cells were co-transfected with cDNAs for mCherry-3xFlag-tagged Orai1 and YFP-STIM1 (0.5 μg each per 35 mm dish). The bath and pipette solution for Orai1 currents contained (in mM) 130 NaCl, 5 KCl, 10 CaCl_2_, 2 MgCl_2_, 10 HEPES and 10 glucose (pH 7.4), and 140 Cs-Asp, 10 BAPTA, 6 EGTA, 6 MgCl_2_ and 10 HEPES (pH 7.2), respectively. Currents were recorded using the whole cell-dialyzed configuration of the patch-clamp technique as described previously [5]. Whole-cell currents were recorded under voltage-clamp using an EPC-9 patch-clamp amplifier (Heka Electronik, Lambrecht, Germany). The patch electrodes were coated with silicone elastomer (Sylgard 184; Dow Corning, Midland, MI, USA), fire-polished, and had resistances of 2–3 MΩ when filled with the pipette solution. The cell membrane capacitance and series resistance were compensated (> 80%) electronically using the EPC9 amplifier. Data acquisition was performed using the PatchMaster software (Heka Electronik). All electrophysiological recordings were performed at room temperature (∼ 20–24 °C).

### Western blot and surface biotinylation assay

Western blotting and cell-surface biotinylation assay as described previously [27]. Briefly, HEK293FT cells were mechanically homogenized in RIPA lysis buffer with protease and phosphatase inhibitors. Primary antibodies were used following as: Orai1 (HPA016583, ATLAS antibodies, Stockholm, Sweden), STIM1 (11565-1-AP, ProteinTech Group Inc., Chicago, IL, USA), GFP (ab137687, Abcam, Cambridge, UK), αKlotho (clone KM2076, KAL-KO603, Cosmo Bio Co., Ltd., Tokyo, Japan), βKlotho (GTX45558, Gene Tex, Inc., Irvine, CA), γKlotho (AF5984-SP, R&D Systems, Minneapolis, MN, USA), Flag-HRP (A8592, Sigma-Aldrich, St. Louis, MO, USA), β-actin (ab6276, abcam, Cambridge, UK), p-Akt^Ser407^ (#9271), p-Akt^Thr308^ (#2965), and Akt (#9272) were provided from Cell Signaling Technology (Beverly, MA, USA). Bands in the immunoblotting were detected and quantified using ChemiDoc XRS+ Imaging System and the ImageLab software (version 5.2.1, *Bio-Rad Laboratories*, Hercules, CA, USA) and the ImageJ software (NIH, USA), respectively. Pretreatment of αKlotho protein and all reagents was processed 1 h before adding serum. Total cellular and biotinylated cell-surface proteins were analyzed by SDS-PAGE followed by western blot. These experiments were performed three times with similar results.

### Confocal microscopy

For immunofluorescence staining, HEK293 cells with an inducible mCherry-STIM1-T2A-Orai1-eGFP were grown on poly-l-lysine-coated coverslips. eGFP-Orai1 and mCherry-STIM1 protein were induced after 12~24 h tetracycline (5 μM) treatment [34] and were fixed with 4% paraformaldehyde in PBS for 15 min at room temperature. GFP and mCherry fluorescent images were obtained using a laser scanning confocal microscope (Zeiss, LSM 800, Jena, Germany) with Airyscan. Super-resolution image of the Orai1 expression on the plasma membrane and Airyscan image processing was acquired using the ZEN 2.3 software.

### In vitro wound-healing assay

A wound-healing assay was conducted as described previously [14, 17]. Briefly, MDA-MB231 and H1693 cells were plated at 1 × 10^7^ per well in a 6-well plate until grown to confluence. The cells were incubated with recombinant αKlotho protein (1 nM) in only 1% penicillin contained RPMI1640 media for 30 min and then exchanged with complete media with or without αKlotho protein. To distinguish cell migration from proliferation, all wound-healing assays were performed in the presence of anti-tumor drug mitomycin C (M4287, Sigma-Aldrich, a final concentration of 0.1 μg/ml) to prevent proliferation. The image was captured by a microscope after 24 h of drug treatment (time 0, initial time point). The migrated cells were counted using an ImageJ 1.48 (NIH, USA).

### Data analysis and statistics

Results are presented as mean ± SEM. Statistical analysis was performed using a two-tailed unpaired Student’s *t* test and one-way ANOVA followed by Tukey’s multiple comparison tests by the GraphPad Prism Software (version 5.0, GraphPad Software, San Diego, CA, USA). *p* values less than 0.05 and 0.01 were considered significant for single and multiple comparisons, respectively. All experiments were repeated independently 3–4 times with similar results.

## Results

### Soluble αKlotho contributes to SOCE regulation

**Fig. 1:**
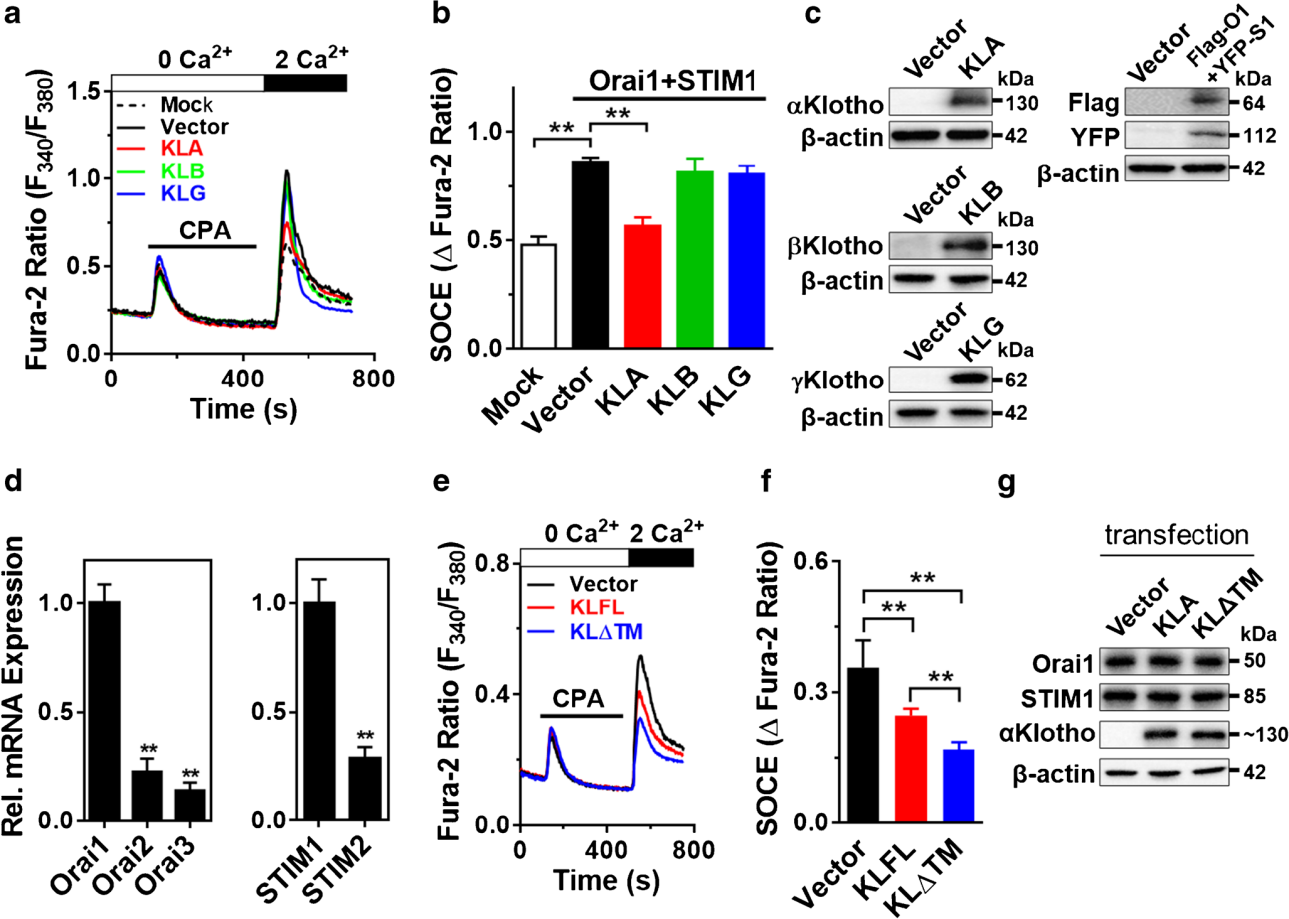
A soluble form of αKlotho downregulates SOCE without affecting Orai1/STIM1. **a** Representative trace showing the effect of the Klotho family on SOCE. Flag-Orai1 and YFP-STIM1 co-transfected with Klotho isoforms: αKlotho (KLA), βKlotho (KLB), or γKlotho (KLG) in HEK293FT cells. Empty vector (pEF1 vector) used as transfection control. **b** Quantification of peak SOCE values is expressed as mean ± SEM (*n* = 55–173 each group). **c** Immunoblotting showing transfection of Klotho isoforms and Flag-tagged Orai1 and YFP-tagged STIM1. **d** Quantitative real-time PCR for relative mRNA expression of Orais and STIMs in HEK293FT cells. **e** Representative trace of endogenous SOCE in HEK293FT cells transiently expressing full-length (KLFL) and secreted (KLΔTM) form of αKlotho. Empty vector (pEF1 vector) was used as a transfection control (Vector). **f** Summary of the SOCE in panel **e** (*n* = 123–186 each group). **g** Effect of αKlotho (KLFL and KLΔTM) on endogenous Orai1 and STIM1 protein expression in HEK293FT cells. **Denotes *p* < 0.01. Data were analyzed by one-way ANOVA (**b** left panel in **d** and **f**) and *t* test (right panel in **d)**

Orai1 and STIM1 couple are the canonical components of SOCE. There are three isoforms in the Klotho family: α, β, and γKlotho [19, 20]. We firstly explored which Klotho isoform regulates Orai1-induced SOCE using HEK293FT cells heterologously expressing Klotho isoforms with Orai1 and STIM1. Overexpression of Orai1 and STIM1 increased SOCE (Fig. 1a–c). Full-length αKlotho inhibited SOCE whereas βKlotho or γKlotho did not affect it (Fig. 1a–c). Among isoforms of Orai and STIM, Orai1 and STIM1 were predominantly expressed in HEK293 cells (Fig. 1d). In following SOCE and immunoblotting experiments throughout the paper, endogenous Orai1 and STIM1 were evaluated.

There are at least two types of functional αKlotho, membranous and soluble form [12]. We next examined which functional mode of αKlotho effectively regulates endogenous SOCE in HEK293FT cells. We found that both membranous and soluble αKlotho downregulate SOCE (Fig. 1e–f). The soluble form of αKlotho is more potent to suppress SOCE. Of note, overexpression of both membranous and secreted forms of αKlotho did not affect the expression of endogenous Orai1 and STIM1 in HEK293 cells (Fig. 1g). Together, soluble αKlotho is critical for SOCE regulation.

### Soluble αKlotho downregulates serum-stimulated SOCE and CRAC current

**Fig. 2:**
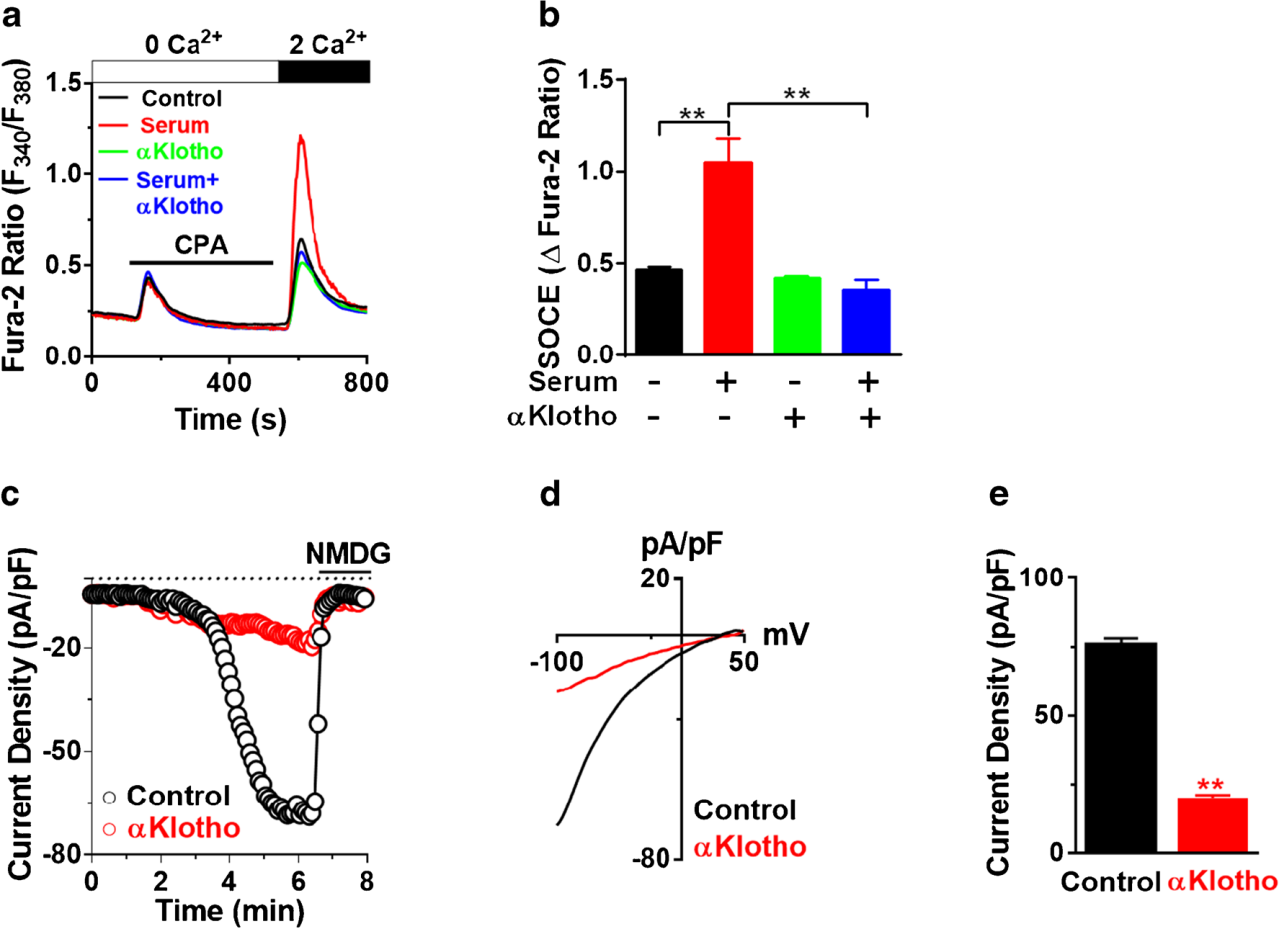
Soluble αKlotho downregulates serum-stimulated SOCE and CRAC current. **a** Representative trace of SOCE showing the effect of soluble αKlotho protein on serum-stimulated native SOCE in HEK293FT. Serum was deprived (SD) for 16 h followed by incubation of serum (10%) with/without recombinant αKlotho protein (1 nM) for 1 h. **b** Summary of the SOCE in panel **a** (*n* = 58–86 each group). **c***-***e** Effect of soluble αKlotho on CRAC channel current density. Time course (**c**), the current-voltage (I-V) relationship (**d**), and current density (**e** *n* = 13–24 each) of CRAC channel current measured under dialyzed whole-cell patch-clamp configuration. All CRAC channel current was measured in HEK293FT cells heterologously expressing Orai1 and STIM1. Orai1 current density was at − 100 mV in **d** and **e**. **Denotes *p* < 0.01. Data were analyzed by one-way ANOVA in **b** and Student’s *t* test in **e**

Soluble αKlotho has pleiotropic cellular function including regulation of ion channels and growth factor signaling [12, 13, 19]. Here, we examined whether soluble αKlotho regulates serum-stimulated SOCE and Ca^2+^ release-activated Ca^2+^ (CRAC) channel current in HEK293FT cells. Endogenous SOCE was significantly increased in the application of serum compared with that in serum-deprived conditions, and this stimulation was attenuated by pretreatment with recombinant αKlotho protein (Fig. 2a and b).

Orai1 is a principal pore subunit of the CRAC channel [33]. Next, CRAC channel current density was measured in HEK293FT cells overexpressing Orai1 and STIM1 by ruptured whole-cell patch-clamp recording. ER Ca^2+^ depletion evoked inward currents under dialyzed whole-cell configuration (Fig. 2c). Current-voltage (*I-V*) relationship curves showed characteristic inward rectifying CRAC currents (Fig. 2d). Soluble αKlotho reduced Orai1 current density and SOCE but had no apparent effects on the general properties of whole-cell currents (Fig. 2c–e). These results support that soluble αKlotho downregulates serum-stimulated SOCE and Orai1 currents.

### Serum increases the cell-surface abundance of Orai1 via stimulating its exocytosis

**Fig. 3:**
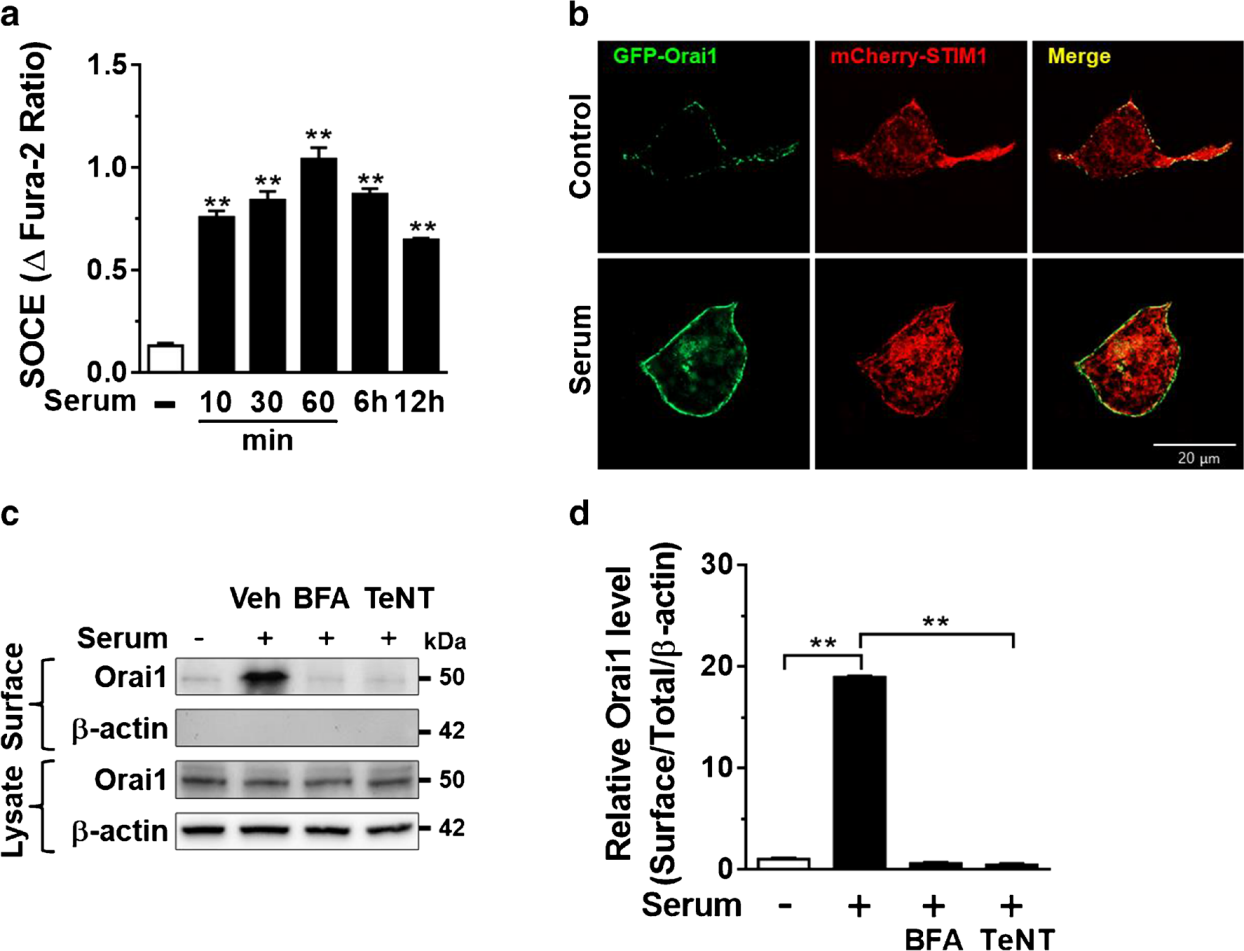
Serum increases the cell surface abundance of Orai1 via stimulating exocytosis of the channel. **a** Time-dependent response of serum incubation on endogenous SOCE (*n* = 47–72 each point). **p < 0.01 vs. Serum deprivation (18 h). **b** Effect of serum (10%, 1 h) on plasma membrane localization of Orai1 in HEK293 cells with an inducible eGFP-Orai1 and mCherry-STIM1 protein. GFP and mCherry signals were measured using a confocal microscope. **c** Representative immunoblotting showing the effect of brefeldin A (BFA, 10 μM for 8 h) or tetanus toxin (TeNT, 60 nM, for 16 h) on the serum-stimulated cell-surface abundance of Orai1 analyzed by biotinylation assay. The lack of β-actin detection in the membrane fraction was used as a control for biotinylation. Surface and lysate denote biotinylated fraction and total cellular protein, respectively. **d** Densitometry of the surface abundance of Orai1 in panel **c**. **p < 0.01, data were analyzed by one-way ANOVA in **a** and **d**

We examined the time course of SOCE stimulation by serum treatment. The stimulation of endogenous SOCE by serum was detected after 10 min incubation and reached a maximal effect at 1 h (Fig. 3a). We and others reported that serum growth factors promote transient translocation of TRPC5 and TRPC6 channels to the plasma membrane [3, 16, 42]. Similarly, serum treatment promoted the relocalization of GFP-tagged Orai1 to the plasma membrane (Fig. 3b). Moreover, biotinylation assay showed that incubation with serum increased the steady-state surface abundance of Orai1 but not in the total cell lysates (Fig. 3c). Assessment of the cell-surface abundance of Orai1 was confirmed by no detection of intracellular protein at a biotinylated fraction (Fig. 3c). The growth factor stimulates a cell-surface abundance of TRPC channels via their SNARE-dependent vesicular exocytosis [9, 16, 43]. Thus, we examined whether a similar mechanism may involve the upregulation of Orai1 by serum. Brefeldin A (BFA) or tetanus toxin (TeNT) disrupt vesicular exocytosis. Serum-stimulated cell-surface abundance of Orai1 was blunted by preincubation with BFA or TeNT (Fig. 3c and d), indicating that steady-state vesicular exocytosis of Orai1 occurs in the presence of serum.

### αKlotho reduces the cell-surface abundance of Orai1 via inhibiting exocytosis of the channel

**Fig. 4:**
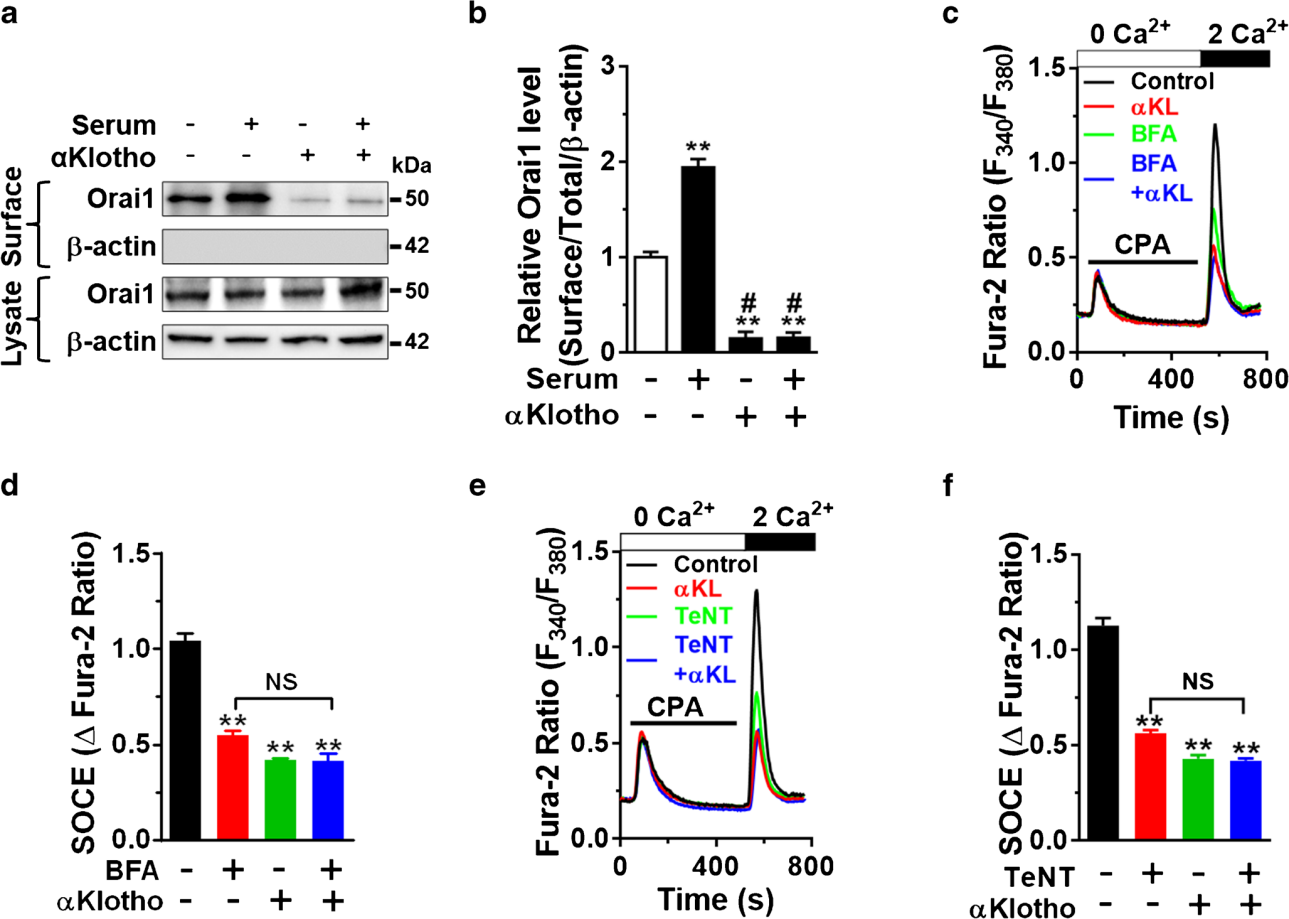
αKlotho downregulates the serum-stimulated cell surface abundance of Orai1. **a** Cell-surface biotinylation assay showing the effect of αKlotho on the serum-stimulated cell-surface abundance of Orai1. **b** Quantification of the results in panel **a**. **p < 0.01 vs. serum deprivation and #*p* < 0.01 vs. serum incubation. **c** Representative SOCE traces showing that αKlotho suppressed SOCE, and prevented the inhibition by Brefeldin A (BFA). Cells were preincubated with BFA (10 μM for 8 h) before αKlotho treatment (1 h). **d** Summary of SOCE in panel **c** (*n* = 53–252 for each). **p < 0.01 vs. vehicle control (no αKlotho). NS, not significant between each group. **e** Representative SOCE traces show that αKlotho reduced SOCE and prevented the suppression by tetanus toxin (TeNT, 60 nM, 16 h). **f** Summary of SOCE in panel **e** (*n* = 84–241 for each). **p < 0.01 vs. vehicle control (no αKlotho). NS, not significant between each group. One-way ANOVA in **b**, **d**, and **f**

We previously reported that soluble αKlotho downregulates cell-surface abundance of TRPC6 in cardiac myocyte and podocyte by inhibiting serum growth factor-dependent exocytosis of the channel [16, 42]. We explored whether a similar mechanism may contribute to the suppression of Orai1 and SOCE. We next measured the effects of αKlotho on a cell-surface abundance of Orai1 using biotinylation assay. Preincubation of soluble αKlotho prevented steady-state and serum-stimulated surface abundance of Orai1 (Fig. 4a and b), which supports the notion that αKlotho reduces the cell-surface abundance of Orai1. These findings of plasma membrane expression of Orai1 were confirmed by Ca^2+^ imaging showing that SOCE was inhibited by BFA or TeNT (Fig. 4c–f). The reduction in the cell-surface abundance of Orai1 by soluble αKlotho may result from decreased exocytosis and/or increased endocytosis of the channel. Moreover, inhibition of vesicular exocytosis of the channel by BFA or TeNT decreased SOCE and prevented further inhibition by soluble αKlotho (Fig. 4c–f). These results indicate that αKlotho reduces SOCE via downregulating vesicular exocytosis of the Orai1 channel.

### αKlotho inhibits SOCE and cell-surface abundance of Orai1 via PI3K-dependent pathway

**Fig. 5:**
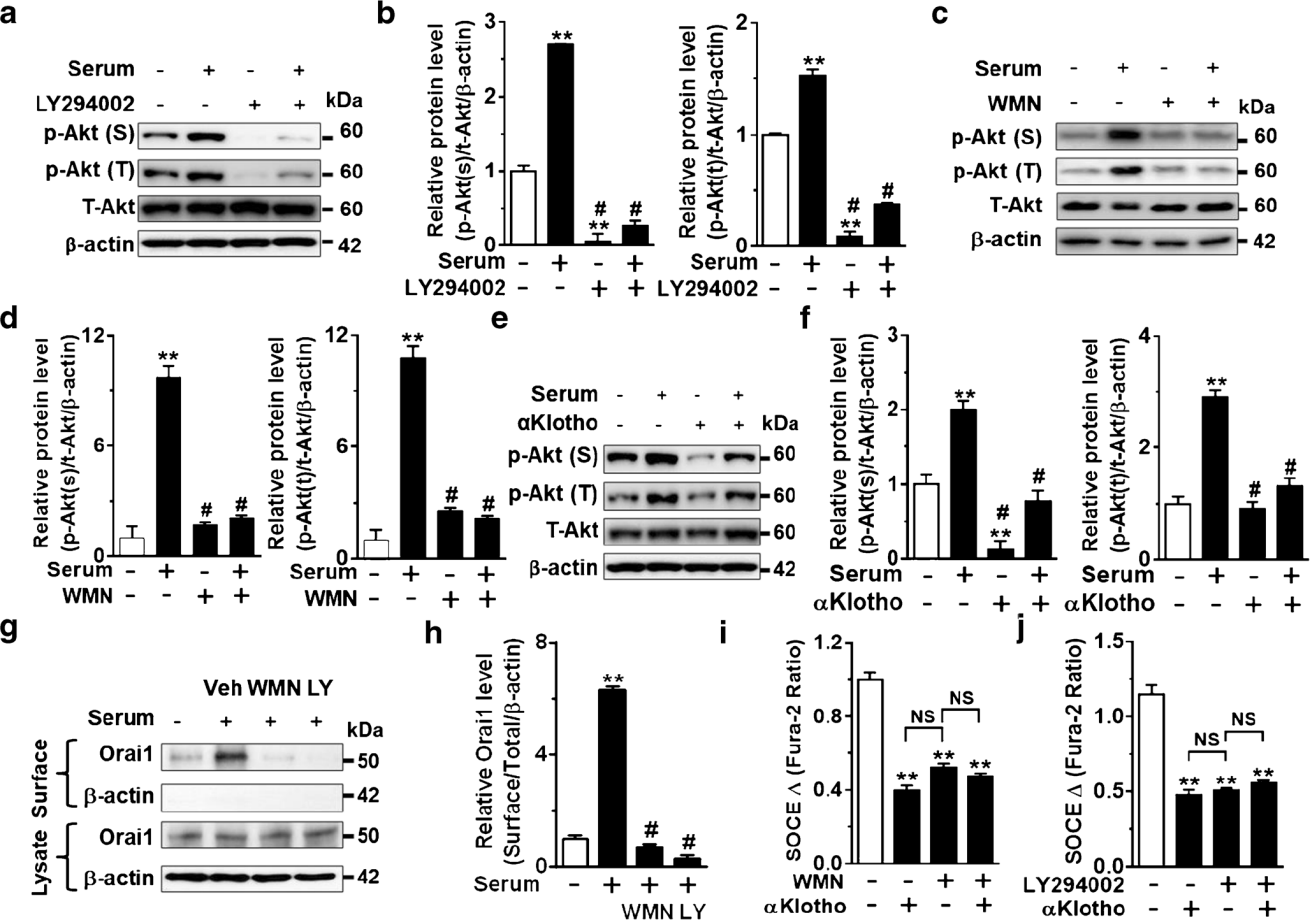
αKlotho inhibits SOCE and cell membrane abundance of Orai1 via the PI3K-dependent signaling pathway. **a**, **c**, and **e** Representative immunoblotting showing that effect of preincubation of PI3K inhibitors. **a** LY294002 (LY, 10 μM for 1 h) and **c** wortmannin (WMN, 50 nM for 1 h) and **e** recombinant αKlotho protein (1 nM for 1 h) on Akt phosphorylation at serine473 (p-Akt (S)) and threonine308 (p-Akt (T)) by serum stimulation (10%, 1 h). **b**, **d**, and **f** Quantification of Akt phosphorylation levels in panel **a**, **c**, and **e** respectively. **p < 0.01 vs. serum deprivation (SD) and #*p* < 0.01 vs. serum incubation. **g** Representative biotinylation assay showing the effect of PI3K inhibitors (WMN and LY) on the cell-surface expression of Orai1 by serum stimulation. **h** Summary of the surface Orai1 level in panel **g**. **p < 0.01 vs. SD and #*p* < 0.01 vs. serum treated. **i** and **j** Summary of SOCE traces showing that αKlotho suppressed SOCE and prevented the inhibition by preincubation of PI3K inhibitors, wortmannin (WMN, *n* = 40–186 each group) or LY294002 (LY, *n* = 76–188 each group), respectively. **p < 0.01 vs. vehicle. NS, not significant between each group. Data were analyzed by one-way ANOVA in **b**, **d**, **f**, and **h–j**

Activation of the PI3K-Akt pathway by serum growth factors increases the plasma membrane abundance of TRPC channels by stimulating their exocytosis [3, 16, 42]. Soluble αKlotho inhibits increased cell-surface abundance of TRPC3 and TRPC6 by inhibiting the PI3K-dependent pathway [9, 16, 42]. We next examined whether αKlotho suppresses SOCE and cell-surface abundance of Orai1 by inhibiting serum-stimulated PI3K signaling. Inhibition of PI3K by preincubation with its blockers, wortmannin (WMN) or LY294002, reduced Akt phosphorylation (Fig. 5a–d). Accordingly, αKlotho also reduced serum-stimulated Akt phosphorylation (Fig. 5e and f). Blockade of PI3K by preincubation with WMN or LY294002 inhibited serum-stimulated cell-surface abundance of Orai1 (Fig. 5g and h). Moreover, inhibition of PI3K by WMN or LY294002 abrogated SOCE and prevented further αKlotho-induced inhibition (Fig. 5i and j). Collectively, these results support that soluble αKlotho suppresses SOCE via inhibiting PI3K-dependent exocytosis of the Orai1 channel.

### αKlotho ameliorates serum-stimulated SOCE and migration in breast and lung cancer cells

**Fig. 6:**
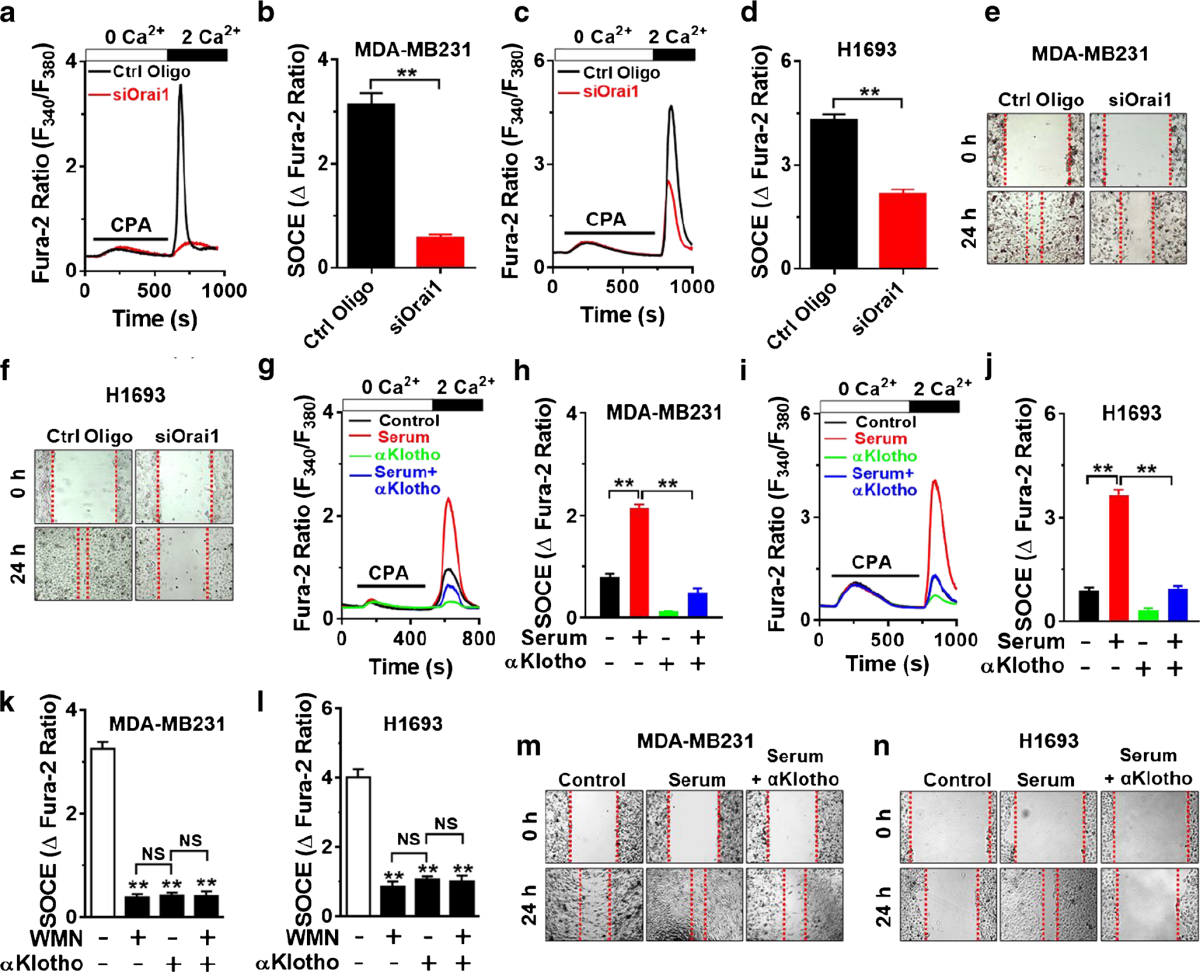
αKlotho suppresses serum-stimulated SOCE and cell migration in breast and lung cancer cells via a PI3K-dependent pathway. **a** and **c** Effect of Orai1 silencing (siOrai1) on SOCE traces in the breast (MDA-MB231) and lung (H1693) cancer cells, respectively. Ctrl Oligo (control oligonucleotide), non-targeting control siRNAs. **b** and **d** Summary of the SOCE in panel **a** and **c**, respectively. **e** and **f** Effect of Orai1 knockdown (siOrai1) on MDA-MB231 and H1693 cell migration, respectively. **g** and **i** Representative SOCE traces showing the effect of soluble αKlotho protein (1 nM for 1 h) on serum-stimulated SOCE in MDA-MB231 and H1693 cells, respectively. **h** and **j** Summary of the results in the panel **g** and **i**. **k** and **l** Effect of soluble αKlotho and PI3K inhibitor, wortmannin (WMN, 50 nM for 1 h, *n* = 104–196 each group) on SOCE in MDA-MB231 and H1693 cells, respectively. **p < 0.01 vs. vehicle. NS, not significant between each group. **m** and **n** Effect of soluble αKlotho on serum-stimulated cell migration of MDA-MB231 and H1693 cells, respectively. Data were analyzed by Student’s *t* test in **b** and **d** and one-way ANOVA in **h**, **j**, **k**, and **l**

Orai1-mediated SOCE is critical for tumor cell migration and metastasis [14, 44]. We previously reported that soluble αKlotho inhibits the migration of clear cell renal cell carcinoma via suppressing the IGF-1-stimulated PI3K pathway [15]. Therefore, we explored whether αKlotho inhibits Orai1-mediated SOCE and migration in breast and lung cancer cells. Orai1 was a primary molecular component of SOCE in both breast and lung cancer cells, MDA-MB231 and H1693, respectively (Fig. 6a–d). Moreover, both tumor cell migrations were also blunted by silencing *ORAI1* (Fig. 6e and f). Consistent with results in HEK293FT cells, SOCE was significantly upregulated by application of serum compared with that by serum deprivation, and the stimulation was attenuated by incubation of αKlotho protein in both MDA-MB231 and H1693 cells (Fig. 6g–j). Of note, inhibition of PI3K by WMN abrogated SOCE and prevented further αKlotho-mediated inhibition (Fig. 6k and l) in both tumor cells supporting the notion that soluble αKlotho suppresses SOCE via inhibiting PI3K-dependent pathway. Accordingly, αKlotho also inhibited serum-stimulated tumor cell migration (Fig. 6m and n).

## Discussion

SOCE is essential for the maintenance of ER Ca^2+^ stores at a precise level for cellular signaling and functions [31, 32]. Disturbed SOCE-mediated Ca^2+^ signaling and homeostasis of Ca^2+^ store have been implicated in the pathogenesis of multiple diseases [31]. Major downstream signaling effectors of growth factor receptors are PLCγ and PI3K-Akt pathways. The cellular mechanism of Orai1 activation can be mediated by serum and/or growth factors triggering PLCγ activation, IP_3_ generation, and Ca^2+^ release from the ER store. Depletion of ER Ca^2+^ store oligomerizes STIM1 to open the Orai1 channel at the plasma membrane [28]. PI3K-Akt pathway signaling contributes to the stimulation of exocytosis of multiple channels such as TRPC5 and TRPC6 [3, 9, 16, 42]. The underlying mechanism of Orai1 regulation by PI3K-derived growth factor signaling remains unsolved. Our data demonstrate that activation of the PI3K-dependent signaling pathway by serum increases the cell-surface abundance of Orai1 via enhancing forward trafficking of the channel to the plasma membrane. These findings support that a similar mechanism may contribute to the downregulation of Orai1-mediated SOCE. Notably, PI3K inhibitors have pleiotropic effects. Therefore, the underlying mechanism by downstream effectors of PI3K to regulate the cell-surface expression of Orai1 awaits future study.

The aging process is closely related to altered growth factor signaling and ion imbalance including Ca^2+^ and P_*i*_ [18, 19]. The membrane-bound form of αKlotho and βKlotho forms a binary complex with FGFRs, which serves as the physiological receptors for FGF23 and FGF19/21, respectively [7, 19–21]. We found that membranous αKlotho but not βKlotho or γKlotho downregulates SOCE. Membranous αKlotho associated with FGF receptors functions as a coreceptor for FGF23 signaling to regulate P_*i*_ [7, 24, 36]. Soluble αKlotho also regulates multiple ion channels [13]. Our data demonstrate that both types of αKlotho can downregulate SOCE. Soluble αKlotho is more potent to downregulate SOCE, supporting that soluble αKlotho is critical for Orai1-mediated SOCE.

Soluble αKlotho can up- or downregulate multiple channels via a distinct mechanism. αKlotho positively regulates several TRPV (TRPV2, 5, and 6) and K^+^ channels (ROMK, Kv1.3, KCNQ1/KCNE1, and hERG channels) through increasing cell-surface abundance of the channels by modification of their *N*-glycan through sialidase or β-glucuronidase activity of αKlotho [1, 2, 6, 26, 27, 29]. Modifying *N*-glycans of the channel by αKlotho delays its endocytosis resulting in increased cell-surface abundance [4, 27]. Conversely, αKlotho negatively regulates multiple TRPC channels with a distinct mechanism. αKlotho directly binds to the VEGFR2/TRPC1 complex to promote their cointernalization [25]. On the other hand, αKlotho downregulates the cell-surface abundance of TRPC6 and TRPC3 via inhibiting their PI3K-dependent exocytosis [16, 42]. In the present study, αKlotho reduces the cell-surface abundance of Orai1 by inhibiting the serum-stimulated PI3K-dependent pathway. This supports the notion that the growth factor-driven PI3K pathway is the downstream effector signaling of soluble αKlotho to regulate Orai1 as well as TRPC3 and TRPC6.

Recently, the underlying mechanism of αKlotho on the downregulation of TRPC6 by growth factor-mediated PI3K signaling is unraveled [9, 41]. Soluble αKlotho specifically targets α2-3-sialyllactose of monosialogangliosides highly enriched in the lipid raft and particularly downregulates lipid raft-dependent PI3K-Akt signaling to suppress TRPC6 [9, 40, 41]. Orai1 is localized in the lipid raft and binds directly to caveolin-1 and cholesterol [10, 45, 46]. At a steady-state, Orai1 continuously recycles between the endosome and the plasma membrane [45, 46]. In the present study, we show that soluble αKlotho suppresses Orai1 surface abundance via inhibiting PI3K-dependent exocytosis of the channel. Hence, future studies will explore the mechanism that specific lipid raft-dependent PI3K/Akt signaling may contribute to the downregulation of Orai1 by soluble αKlotho.

Accumulating evidence demonstrates that the upregulation of Orai1/STIM1-mediated SOCE is associated with tumor progression and poor prognosis in multiple cancers including breast, lung, and renal cancer [14, 35, 44]. Hyperactivation of the PI3K/Akt signaling pathway promotes tumor cell migration. Currently, targeting growth factor receptor-driven PI3K signaling pathway with pharmaceutical agents have been suggested as a therapeutic solution for treating cancers and applied in clinical trials [8, 37, 38]. αKlotho suppresses growth factor-stimulated cell migration by inhibiting PI3K/Akt pathway in multiple tumors such as breast and renal cancers [15, 39]. This study provides compelling evidence supporting αKlotho targeting PI3K-stimulated SOCE function as a tumor suppressor. SOCE is critical for finetuning ER Ca^2+^ stores for cellular signaling and function and its altered activity leads to pathologies [31, 32]. Hence, αKlotho-based approaches may be attractive targets for treating SOCE-related pathologies including tumors.

## Abbreviations

ANOVA: analysis of variance
BFA: Brefeldin A
cDNA: complementary DNA
CPA: cyclopiazonic acid
CRAC: calcium release-activated calcium
ER: endoplasmic reticulum
FBS: fetal bovine serum
FGF: fibroblast growth factor
FGFR: fibroblast growth factor receptor
hERG: Ether-a-go-go-related gene
Kv1.3: voltage-gated potassium channel, shaker-related subfamily, member 3
KCNQ1: voltage-gated potassium channel subfamily Q member 1
KCNE1: voltage-gated potassium channel subfamily E regulatory subunit 1
KLFL: transmembrane full-length mouse αKlotho
KL△TM: Extracellular domain of mouse αKlotho
GFP: Green fluorescent protein
*Lctl*: lactase-like
P_*i*_: inorganic phosphate
PI3K: phosphoinositide-3-kinase
PLCγ: phospholipase C gamma
IP_3_: inositol trisphosphate
RIPA: radioimmunoprecipitation assay
ROMK: renal outer medullary potassium channel
SDS-PAGE: sodium dodecyl sulfate-polyacrylamide gel electrophoresis
SEM: standard error of the mean
SNARE: SNAP receptor
STIM1: stromal interaction molecule 1
SOCE: store-operated Ca^2+^ entry
TeNT: tetanus toxin A
TRP: transient receptor potential
TRPC: transient receptor potential canonical type
TRPV: transient receptor potential vanilloid type
VEGFR: vascular endothelial growth factor receptor
WMN: Wortmannin
YFP: Yellow fluorescent protein
